# Validity and Reliability of the Turkish Version of the My Family′s Accessibility and Community Engagement (MyFACE) Tool

**DOI:** 10.1155/oti/9345859

**Published:** 2026-08-14

**Authors:** Ayşe Göktaş, Esma Özkan, Tuana Harman

**Affiliations:** ^1^ Department of Occupational Theraphy, Gülhane Faculty of Health Sciences, University of Health Sciences, Keçiören, Ankara, Türkiye, akdeniz.edu.tr; ^2^ Department of Occupational Theraphy, Gülhane Institute of Health Sciences, University of Health Sciences, Keçiören, Ankara, Türkiye, akdeniz.edu.tr

**Keywords:** child and adolescent participation, children with disabilities, family participation, My Family′s Accessibility and Community Engagement, Turkish version

## Abstract

**Background:**

Community participation and environmental accessibility are core to inclusive practices for children with disabilities and their families.

**Objective:**

The aim was to examine the Turkish validity and reliability of My Family′s Accessibility and Community Engagement (MyFACE) Scale. It was conducted with the parents and caregivers of 105 children with disabilities who continue to receive education in special education and rehabilitation centers.

**Methods:**

Data was obtained using the Participation Scale (PS), Child and Adolescent Participation Scale (CAPS), and the MyFACE.

**Results:**

Cronbach′s alpha internal consistency coefficient for total scale was found to be 0.859. Through MRFA performed to determine the number of subdimensions of the MyFACE scale, a unidimensional structure consisting of nine items was obtained. The factor loadings of the items forming the single dimension ranged from 0.584 to 0.865. It was found that the factor loadings of the scale can be classified as ranging from good to excellent. According to the results of the analysis, the *χ*2 statistic (*χ*2 = 36.857, *p* = 0.077), *χ*2/*s* *d* value (0 < 1.418 ≤ 2) and fit indices TLI (0.959), CFI (0.971), GFI (0.921), and RMSEA (0.063) evaluated according to cutoff points in literature showed good fit between the model and the data set. The Cronbach alpha values for these tests were 0.894 and 0.887, respectively. When the scale was readministered to 48 participants after a certain period, the intraclass correlation coefficient (ICC) was found to be 0.980 (ICC = 0.980, *p* < 0.001).

**Conclusions:**

The Turkish version of the MyFACE tool can be used in clinical settings or research alongside any intervention aimed at enhancing children′s participation and can help support families′ participation in the community.

## 1. Introduction

### 1.1. Community Participation and Accessibility in Families of Children With Disabilities

Community accessibility and participation are essential elements of engagement for individuals with disabilities and their families [1]. Parents′ perceptions of accessibility significantly influence the community involvement experiences of children with disabilities [2, 3]. The International Classification of Functioning, Disability and Health (ICF) states that children′s activities and social involvement are influenced by individual health status, functional capacity, as well as environmental and personal factors [4]. From the viewpoint of parents with impaired children, societal involvement is affected by complex environmental elements, including the accessibility of physical spaces, social attitudes, service systems, and regulatory frameworks. Research indicates that children′s engagement is significantly affected by their capabilities, parental support, financial resources, and the availability of community infrastructure [5]. Law et al. identified that parents face physical, structural, and social obstacles regarding their children′s engagement in educational, communal, and recreational activities [6]. In the extensive review by Piškur et al., it was highlighted that parents needed to formulate diverse strategies to facilitate their children′s active engagement in daily life; nonetheless, they encountered obstacles stemming from environmental limitations, insufficient information, and systemic deficiencies [7]. Shields and Synnot illustrated that obstacles from both the physical and social environments restrict children′s engagement in the process of parental guidance toward physical activity [8]. These findings underscore, as highlighted by the ICF, the imperative for supportive systems at the environmental and policy levels in addition to human interventions [4] Mothers of children with disabilities encounter financial strain, physical exhaustion, and psychological difficulties to facilitate their children′s access to social resources [9, 10]. Goudie et al. highlighted that caregiving responsibilities for families with handicapped children correlate with significant financial strain due to diminished workforce engagement [9]. Furthermore, a qualitative study revealed that women encountered “insufficient inclusive public spaces, challenging transportation conditions, inadequate working hours,” and social isolation [10]. These problems adversely affect the mental health of mothers, resulting in symptoms such as burnout, a predisposition to depression, and disrupted sleep [11, 12]. Consequently, these findings indicate that families with impaired children necessitate supportive mechanisms at both individual and societal levels to alleviate the challenges of caregiving, financial hardships, and obstacles to social engagement [3].

### 1.2. Impact of Community Engagement on Family Well‐being

Community engagement is a crucial element influencing family well‐being, resilience, and quality of life, enabling engagement in supportive networks and access to community resources.^13^ Research indicates that engaged family involvement in social and community activities correlates with less caregiver load and enhanced mental health outcomes [2]. Furthermore, inclusive community initiatives and accessible surroundings are crucial for enhancing the overall engagement and life satisfaction of children with disabilities and their families [5]. The adoption of family‐centered policies and programs that promote social involvement can mitigate the adverse impacts of isolation and empower families to more efficiently confront the challenges of raising a child with disabilities [3].

### 1.3. Limitations of Existing Measures in Assessing Parent‐Centered Participation and the My Family′s Accessibility and Community Engagement (MyFACE) Scale

Parental health and well‐being significantly influence children′s participation and social engagement, underscoring the necessity of a comprehensive approach to community accessibility research [13, 14]. Established instruments like the Family Quality of Life Scale (FQOL) [15], Craig Hospital Inventory of Environmental Factors (CHIEF for Children–Parent Version) [16], and Community Integration Questionnaire (CIQ) [17] serve as effective measures for assessing families′ overall quality of life, environmental impediments, and degrees of social integration. However, they appear inadequate for explicitly assessing the social engagement experiences of parents with handicapped children. The FQOL scale offers significant insights across five subdimensions (family interaction, parenting, emotional well‐being, physical/material well‐being, and level of special support), yet it lacks direct enquiries about parents′ social activities and community roles [18, 19]. Likewise, the parent‐adapted CHIEF for Children‐Parent Version identifies child‐centered environmental pressures by assessing physical, structural, and policy barriers, yet it neglects to examine the individual responses of parents to these barriers or the importance of social participation opportunities from the parent′s viewpoint [16]. The CIQ evaluates household, social, and productive activities through 15 items, focusing on the overall behavioral participation of adults or family members, yet it inadequately exposes the participation constraints faced by parents of children with disabilities in their anticipated supportive roles [17, 20]. This situation unequivocally illustrates the necessity for instruments that directly evaluate the obligation of parents to reconcile their specialized caring responsibilities with their social lives. Consequently, in formulating policies and programs to enhance social involvement, it is essential to broaden the measurement framework with novel parent‐centered measures or supplementary modules to rectify this deficiency. Nonetheless, there is an absence of standardized instruments to accurately assess these perceptions [3]. MyFACE scale was created to fill this gap and offer a means to evaluate how families of children with disabilities view community access and involvement [3]. MyFACE offers insights into families with a disabled child and the obstacles recognized by the family. It readily recognizes obstacles including parental health and weariness, perceived stigma, physical inaccessibility, insufficiently qualified help, transportation difficulties, and time constraints.

### 1.4. Cross‐Cultural Adaptation and Global Relevance of MyFACE

In addition to its initial development, understanding the cross‐cultural applicability of measurement tools is essential to ensure their relevance across diverse sociocultural contexts. The MyFACE scale was originally developed and psychometrically evaluated in Australia, demonstrating satisfactory content validity, construct validity, and internal consistency in mothers of children with disabilities [2, 3]. Subsequent studies have confirmed its unidimensional structure and acceptable reliability, highlighting its capacity to capture a unique construct related to parental perceptions of community accessibility and engagement [2]. However, despite these promising findings, the global use and cross‐cultural adaptation of the MyFACE scale remain limited. This is noteworthy given that perceptions of accessibility and community participation are highly context‐dependent and influenced by cultural norms, environmental structures, and social policies.

In the broader rehabilitation and occupational therapy literature, cross‐cultural adaptation studies of family‐centered instruments consistently emphasize the need for rigorous translation, cultural equivalence, and psychometric validation to ensure conceptual and measurement validity across populations [21, 22]. Therefore, adapting MyFACE into different cultural contexts is crucial not only to confirm its structural and functional validity but also to examine its sensitivity to culturally specific barriers and facilitators of community participation. Expanding the evidence base through such studies will enhance the global applicability of the tool and contribute to a more comprehensive understanding of family experiences in diverse settings.

### 1.5. Cultural Context and Family Structure in the Turkish Context

Understanding the cultural context in which family‐centered constructs are evaluated is essential for accurately interpreting accessibility and community engagement, as the meaning and relevance of such constructs may vary across sociocultural settings [23, 24]. In Turkey, family structure and social life are characterized by collectivist tendencies, interdependence, and extended family involvement, which can significantly shape caregiving roles and community participation experiences. Compared with more individualistic societies, caregiving responsibilities in Turkey are more frequently assumed within the family unit, particularly by mothers, who often play a central role in coordinating care and facilitating children′s participation in daily and social activities [25–28]. Although collectivist family structures may facilitate emotional and instrumental support through close‐knit social networks, they may simultaneously intensify caregiving responsibilities and constrain opportunities for autonomous social participation, particularly among mothers of children with disabilities [25, 28, 29].

In addition, societal attitudes toward disability, the accessibility of public environments, and the inclusiveness of community services are shaped by sociocultural and policy contexts and may therefore vary across countries. Compared with more individualistic societies, Turkey is characterized by stronger family interdependence, close‐knit social networks, and culturally embedded forms of solidarity, which play a central role in shaping community engagement. The strong social ties, neighborhood relations, and culturally embedded solidarity practices may facilitate certain forms of community engagement that are less formal but socially meaningful [30–32]. Within this context, caregiving responsibilities are predominantly assumed within the family, and families often rely on informal support systems alongside formal services when navigating community participation.

At the same time, as in many settings, participation experiences of individuals with disabilities and their families are influenced by environmental determinants such as transportation systems, physical accessibility, and societal attitudes, which have been consistently identified as key barriers to participation across diverse contexts [33, 34]. Although ongoing policy initiatives in Turkey have contributed to improvements in accessibility and inclusion, the interaction between formal service provision and culturally embedded informal support structures may differ from that observed in other countries where family‐centered services are more systematically institutionalized. These characteristics highlight both similarities and differences between Turkey and other cultural contexts where tools like MyFACE have been developed and applied.

### 1.6. Rationale for the Present Study

These contextual characteristics highlight both shared and culturally specific aspects of community engagement, underscoring the importance of examining how perceptions of accessibility and participation are experienced and measured within the Turkish context. Such an approach is essential to determine whether the constructs assessed by the MyFACE scale are interpreted similarly across cultures or are shaped by culturally specific dynamics. Considering that parental support and advocacy significantly impact children′s anticipations of social engagement, evaluating the validity and reliability of a Turkish‐translated version of MyFACE will yield crucial insights for scholars and practitioners in rehabilitation and inclusive policy development. This research seeks to enhance global discourse on accessibility and community engagement by translating and validating the scale, thereby offering an evidence‐based instrument to guide actions and policy suggestions.

## 2. Materials and Methods

### 2.1. Design

The study was conducted in Special Education and Rehabilitation Centers located in Ankara. A total of 105 volunteer caregivers were included in the study. The research was carried out with the caregivers of 105 preschool children attending seven different classes in two separate special education institutions affiliated with the Ankara Provincial Directorate of National Education, which were selected using a simple random sampling method.

A quantitative research design was employed. Although 150 caregivers of students were invited to participate in the study, 105 caregivers agreed to take part. Participants were included in a face‐to‐face questionnaire assessment conducted in schools affiliated with the Ministry of National Education. Informed consent was obtained from the caregivers of the children for participation. Caregivers were informed about the purpose of the study and provided written consent. Participants were assured of anonymity, and it was stated that the data would be used solely for research purposes. Completion of the questionnaires took approximately 40 min.

Ethical approval was obtained from the non‐Interventional Scientific Research Ethics Committee of the University of Health Sciences.

#### 2.1.1. Procedure

Study was conducted between 30 May 2023 and 1 January 2024. It was initiated after approval from the ethics committee. This study was conducted in accordance with the Declaration of Helsinki. All participants in the study group took part on a voluntary basis. Signed voluntary informed consent forms were obtained from all patients who participated in the study. Informed consent was obtained from the children′s caregivers for participation. The caregivers were informed about the purpose of the study.

Individuals who did not wish to participate were excluded from the assessment. At the beginning of the study, during the data collection process, the MyFACE test was administered face‐to‐face by an occupational therapist specialized in the field. After the child′s session at the special education center, the caregiver and the child were taken to a quiet and calm assessment room, where the caregiver was asked to complete the questionnaire. The demographic information form asked participants about their age, education level, and other demographic data. The assessment was conducted using questionnaires.

#### 2.1.2. Ethical Considerations

The study group was obtained from Health of Sciences University, non‐Interventional Clinical Research Ethics Committee (ethical date 16.05. 2023, Decided Number 2023‐184).

All participants were informed that they could withdraw from the study at any time and that their data could be deleted upon request. The study design minimized the risk of harm to participants, as all data were anonymized and it was impossible for any individual′s information to be disclosed to other participants.

Moreover, since the study involved only the administration of questionnaires, there was no risk of harm to the participants. As the study did not include any intervention or the collection of sensitive personal data, it posed no foreseeable risk to the participants. The potential benefits of the research for society and the participants outweigh any minimal risks, given that the study presented virtually no risk to those involved.

#### 2.1.3. Participants

The study included parents and caregivers of children who needed special education and rehabilitation, such as cerebral palsy, autism spectrum disorder, sensory processing differences, and so on. and who were actively continuing their education in an institution and volunteered to participate in the study. Individuals who had problems reading, understanding, and communicating in Turkish were excluded from the study.

#### 2.1.4. Inclusion Criteria

The inclusion criteria are as follows: (1) accept the consent form; (2) have the ability to communicate; (3) Volunteer to take part in the research; and (4) speak Turkish as their language (shown in the flowchart).

#### 2.1.5. Exclusion Criteria

The participants should be as follows: (1) be able to communicate; (2) did not volunteer to be a part of the research; and (3) have any psychiatric or emotional disorders.

### 2.2. Assessment Tools

Sociodemographic information was taken from all participants. Additionally, participants were administered the Nottingham Health Profile (NHP) [35], Participation Scale (PS) [36], Child and Adolescent Participation Scale (CAPS) [37], and the MyFACE tool [3].

#### 2.2.1. Sociodemographic Information Form

Age, gender, BMI, education level, marital status, diagnosed health condition, child′s disability (intellectual disability, autism spectrum disorder, cerebral palsy, ADHD, sensory processing differences, genetic disability, and other), number of diagnoses for the child, child′s age, use of assistive devices, and so on were recorded.

#### 2.2.2. NHP

We assessed the quality of life using the Turkish version of the NHP. NSP consists of 6 areas: physical mobility (8 items), pain (8 items), sleep (5 items), energy level (3 items), emotional reactions (9 items), and social isolation (5 items). When a “yes” answer is given for any item, the weighted score assigned to it is taken. The total score for an area is determined by summing the weighted scores of the “yes” answers given to that area. The best score for each area is ’0,’ and the worst score is ’100’ [35].

#### 2.2.3. PS

PS, based on the ICF, assesses involvement in domestic life, self‐care, mobility, interpersonal contacts and relationships, engagement in paid or voluntary employment, and participation in community and social activities. The individual responds to the inquiry by juxtaposing themselves with their contemporaries. If they perceive themselves as analogous to their peers concerning the posed subject, they receive a score of “0” and go to the subsequent question. It comprises 18 questions. If the answer is negative, the inquiries proceed according to the degree of the issue encountered: “1” point is awarded for no problem; 2 points for a minor problem; 3 points for a severe problem; and 5 points for a major problem (minimum 0–maximum 90 points). A high score signifies significant participation limits [36].

#### 2.2.4. CAPS

This assessment evaluates children′s engagement in domestic, educational, and communal activities based on data supplied by the family [37, 38]. The instrument comprises 20 items divided into four subsections: home participation (6 items), neighborhood and community participation (4 items), school participation (5 items), and home and community life activities (5 items). This assessment is completed by the child′s caregiver or family member. The assessment is performed on a scale of 100 points. Low ratings signify minimal participation, whereas high levels denote substantial participation [37].

#### 2.2.5. MyFACE Scale

MyFACE evaluates how parents′ perceptions about community involvement and accessibility for families with a disabled child. On a 5‐point Likert scale, items indicate common community activities. Items denote prevalent community activities assessed on a 5‐point Likert scale. It assesses the “face” that moms caring for a kid in need exhibit toward the community, along with their past and present experiences of social engagement [2]. MyFACE is a nine‐item instrument that assesses mothers′ perceptions of community accessibility and engagement. It offers insights into families with a disabled child and the obstacles recognized by the family. It readily recognizes obstacles including parental health and weariness, perceived stigma, physical inaccessibility, insufficient qualified help, transportation difficulties, and time constraints. MyFACE is a clinical instrument that pertinent healthcare professionals can utilize to enhance families′ perceptions of accessibility and inclusivity, foster family engagement within their communities, and pinpoint areas necessitating improvement [3].

### 2.3. MyFACE Scale Translation and Adaptation

The researchers who created the scale gave the required authorizations. The test form was administered to 105 subjects, whereas Guillemin′s method was used twice to five expert panelists [39].

The initial version of the scale was translated from English to Turkish by two independent linguistic specialists for linguistic validity. The authors assessed the items of the Turkish version of the scale for adherence to Turkish language conventions. Adjustments were made to the scale in accordance with professional judgments, resulting in the creation of a unified Turkish text. The Turkish version of the scale was independently retranslated into English by two translators. To assess the linguistic quality, clarity, and conceptual fidelity of this Turkish translation of the scale, the insights of four rehabilitation specialists were solicited. The experts were responsible for the consolidation of all questionnaire versions and the development of the prefinal version for field testing. Consequently, the experts conducted a comprehensive evaluation of all the translations and reached consensus regarding any discrepancies. The pilot study was conducted on 10 caregivers selected according to specific inclusion criteria. In addition, a pilot test involving 10 participants was done to evaluate the comprehensibility of the Turkish version of the scale, and feedback was gathered. The scale was finalized based on feedback from the participants and approval for the final Turkish version of the scale was obtained from those who developed the scale. The study on the validity and reliability of the scale commenced with the developed Turkish version [38–41]. For the test‐retest analysis of the study, the forms were completed again by 48 participants after a 15‐day interval.

#### 2.3.1. Sample Size

The number of participants required for the study was calculated as 105 (with medium effect size, Type I error, and 90% power).

### 2.4. Statistics

Mean ± standard deviation was used for continuous data, and frequency and percentage were used as descriptive statistics for categorical data. The Kolmogorov–Smirnov test was used to determine if continuous data conformed to a normal distribution. Multivariate normal distribution was assessed using Mardia′s multivariate kurtosis and skewness statistics [42]. No missing data or outliers were found in the dataset. The presence of multivariate normal distribution was examined using Mardia′s [42] multivariate kurtosis and skewness statistics [43]. As a result of Mardia′s multivariate normality test, the multivariate kurtosis coefficient value (125.922, sd: 165, *p* < 0.001) was statistically significant, indicating that the scale items do not follow a multivariate normal distribution. Due to this result, the most preferred polychoric correlation matrix was used in the exploratory factor analysis (EFA) [44, 45].

Since the determinant value of the polychoric matrix is (0.00347 > 0.00001), it is concluded that there is no multicollinearity problem among the scale items [46]. The discriminatory power of the nine items that make up the MyFACE scale was evaluated using the item analysis based on correlation suggested by Likert [47]. If the item‐total correlation coefficient was positive and greater than 0.30 [48], and if the Cronbach′s alpha value significantly decreased when the item was removed, the item was retained in the scale. The psychometric properties of the scale were evaluated through validity and reliability analyses. The structural validity of the scale items was examined using minimum rank factor analysis (MRFA) [49] and confirmatory factor analysis (CFA).

The suitability of the data structure for factor analysis was determined using the Kaiser–Meyer–Olkin (KMO) test and Bartlett′s test of sphericity. The direct oblimin [50] oblique rotation method was used as the factor rotation technique, whereas the number of dimensions was determined using the minimum average partial (MAP) method, parallel analysis (PA), and the Kaiser (K1) criterion [51]. In CFA, the parameter estimation method used was Maximum Likelihood Estimation (MLE). Model fit was evaluated through the statistical significance of factor coefficients and goodness‐of‐fit indices.

In the evaluation of the scale′s reliability, Cronbach′s alpha (*α*) and McDonald′s omega (*ω*) coefficients were used as internal consistency coefficients, whereas the intraclass correlation coefficient (ICC) was used for test‐retest reliability [47]. An ICC analysis was conducted using a 2‐way mixed‐effects model with the absolute agreement type selected. The obtained results were interpreted according to the cutoff points proposed by Cicchetti: less than 0.40—poor, between 0.40 and 0.59—fair, between 0.60 and 0.74—good, between 0.75 and 1.00—excellent [52].

In the data analysis, Jamovi Ver.1.6.6 was used, FACTOR 11.05.01 for MRFA, and AMOS 21.0 for CFA. Statistical decisions were made at a significance level of 0.05.

## 3. Results

### 3.1. Participants

A total of 105 participants were included in our study. Of the caregiving participants, 95 (90.5%) were women, 10 (9.5%) were men, 99 (94.3%) were married, and the mean age was 38.26 ± 8.61 years. Forty‐one (39.0%) of the caregiving participants had primary school education, 31 (29.5%) had high school education, and 31 (29.5%) had bachelor′s or graduate‐level education (Table 1). In Table 1, sociodemographic data for the children being cared for were examined. It was found that 60 (57.1%) of the children were male, 45 (42.9%) were female, and their mean age was 7.82 ± 0.50 years. Forty‐two (40.0%) of the children attended public schools, 22 (21.0%) attended special education application schools, and 36 (34.3%) did not attend school (Table 1).

**Table 1:** Sociodemographic characteristics of caregivers and children (*N* = 105).

Characteristics		*M* *e* *a* *n* ± *S* *d* (min–max)
Caregiver′s age (mean ± sd)		38.26 ± 8.61 (19–75)
Child′s age (mean ± sd)		7.82 ± 0.50 (1–28)
Characteristics	Category	Frequency *n* (%)
Caregiver′s gender	Woman	95 (%90.5)
	Man	10 (%9.5)
Caregiver′s psychosocial health problem	Yes	5 (%4.8)
	No	100 (%95.2)
Caregiver′s marital Status	Married	99 (%94.3)
	Separated or divorced	6 (%5.7)
Caregiver′s caregiver	Yes	8 (%7.6)
	No	97 (%92.4)

Caregiver′s education level	Illiterate	2 (%1.9)
	Primary school	41 (%39.0)
	High school	31 (%29.5)
	Undergraduate degree and Postgraduate	31 (%29.5)

Caregiver′s work status	Housewife	83 (%79.0)
	Worker	20 (%19.0)
	Retired	2 (%1.9)

Number of children of the caregiver	0	2 (%1.9)
	1	17 (%16.2)
	2	52 (%49.5)
	3	22 (%21.0)
	4 and upper	12 (%11,5)

Child′s gender	Girl	45 (%42.9)
	Boy	60 (%57.1)
Child′s assistive device	Yes	55 (%52.4)
	No	50 (%47.6)

Child′s school category	Public school	42 (%40.0)
	Private School	5 (%4.8)
	Special education practice school	22 (%21.0)
	Does not go to public school	36 (%34.3)

Disorder of children	Mental retardation	5 (%4.76)
	ASD	23 (%21.99
	Cerebral Palsy	42 (%40)
	ADHD	15 (%14.7)
	Sensory processing problems	13 (%12.3)
	Genetic disorders	7 (%6.6)

Abbreviations: ADHD, attention‐deficit/hyperactivity disorder; ASD, autism spectrum disorder.

### 3.2. Item Analysis Results

When examining the item analysis results in Table 2, it was observed that the corrected item‐total correlation coefficients for the items were positive and above 0.30 [48]. A decrease in Cronbach′s alpha was observed when any of the nine items were removed. Therefore, all items were retained in the scale. The Cronbach′s alpha internal consistency coefficient for the total scale was found to be 0.859.

**Table 2:** Item analysis and exploratory factor analysis (EFA) results (*N* = 105).

	Scale items	All average when item is deleted	All variance when item is deleted	Item‐total correlation coefficient	*R* ^2^	Cronbach alpha when item is deleted
Cronbach alpha = 0.859mean ± SD 25.05 ± 7.90	Item 1	22.000	53.885	0.467	0.292	0.854
	Item 2	22.038	51.960	0.502	0.281	0.851
	Item 3	22.228	47.909	0.708	0.643	0.831
	Item 4	22.181	49.438	0.667	0.588	0.836
	Item 5	23.361	47.522	0.556	0.342	0.849
	Item 6	22.219	49.230	0.642	0.666	0.838
	Item 7	22.142	51.258	0.617	0.639	0.841
	Item 8	22.114	49.910	0.597	0.425	0.842
	Item 9	22.095	50.683	0.533	0.329	0.849
EFA results						
Items	Factor 1					
Item 1	0.584					
Item 2	0.589					
Item 3	0.865					
Item 4	0.770					
Item5	0.696					
Item 6	0.817					
Item 7	0.782					
Item 8	0.727					
Item 9	0.684					

*Note:* All factor loadings > 0.50 indicate acceptable construct validity.

In the subsequent process, the psychometric properties of the scale were evaluated through validity and reliability analyses.

### 3.3. Structural Validity Stages

The structural validity of the MyFACE scale was evaluated through EFA and CFA.

### 3.4. EFA Results

Looking at the results in Table 2, the KMO value (0.813) exceeds the cutoff point of 0.80 suggested by Koçak et.al. [51], indicating an excellent level of sample adequacy. The Bartlett′s Test of Sphericity was used to examine whether the polychoric correlation matrix significantly differed from the identity matrix. According to the obtained results (*χ*2 = 388.50, df = 36, *p* < 0.001), it was observed that there was sufficient correlation among the data [53]. These results indicate that the dataset is suitable for applying MRFA.

**Figure 1:**
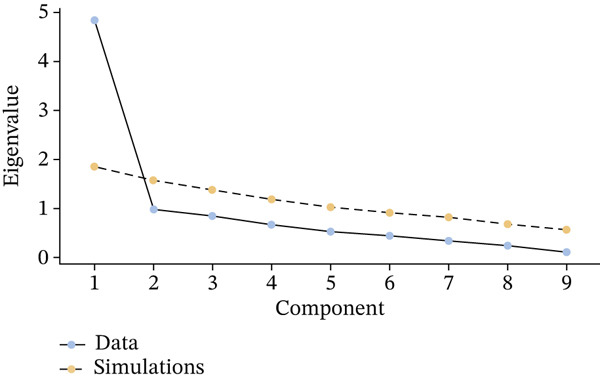
Scree plot.

The values obtained from the PA method and the corresponding scree plot [54] are shown in Figure 1. According to the figure, a unidimensional structure was determined for the scale.

Through MRFA performed to determine the number of subdimensions of the MyFACE scale, a unidimensional structure consisting of nine items was obtained (Table 2). The factor loadings of the items forming the single dimension ranged from 0.584 to 0.865. According to the cutoff points established by Comrey and Lee, it was found that the factor loadings of the scale can be classified as ranging from good to excellent [55].

Additionally, using the reduced correlation matrix, the eigenvalue for the single dimension was found to be 4.79, with the proportion of total common variance explained by the unidimensional factor model being 70.79% (cumulative variance: 70.79; total common variance = 6.766).

### 3.5. CFA Results

**Figure 2:**
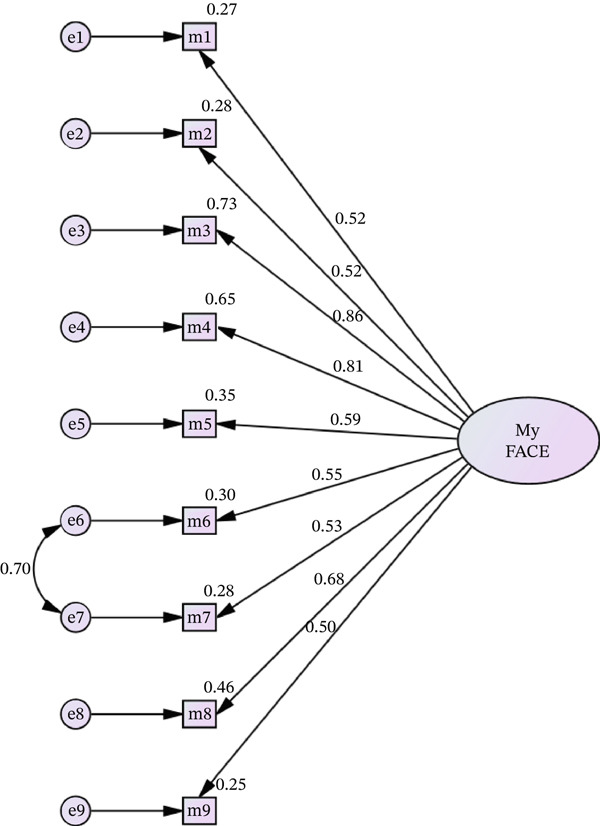
Primary‐level CFA measurement model of the scale.

In Figure 2, the modified one‐dimensional primary‐level CFA measurement model, which includes a modification suggested by the modification index of the scale, is shown. The model′s fit to the data was evaluated in four stages according to the CFA results.

First, the strength, direction, and statistical significance of the factor loading estimates were examined. In the subsequent three stages, the goodness‐of‐fit indices obtained from the CFA results were evaluated [54]. In Table 3, the *t*‐statistics of the unstandardized factor loadings for the nine items of the unidimensional structure are greater than 1.96, indicating statistical significance (*p* < 0.001). The standardized factor loadings range from 0.498 to 0.855. Since the factor loading parameter estimates are statistically significant, the first step in confirming the scale′s unidimensional measurement model has been completed [56].

**Table 3:** Confirmatory factor analysis (CFA) results and model fit indices.

Variable and indicators	Nonstandard factor Loadings	SE	*t*‐value (critical rate)	*p*	Standard factor loadings
Item 1	1				0.519
Item 2	1.161	0.279	4.164	< 0.001	0.525
Item3	2.000	0.375	5.326	< 0.001	0.855
Item 4	1.773	0.337	5.258	< 0.001	0.809
Item 5	1.694	0.386	4.392	< 0.001	0.589
Item 6	1.252	0.296	4.232	< 0.001	0.546
Item 7	1.056	0.257	4.107	< 0.001	0.526
Item 8	1.562	0.329	4.746	< 0.001	0.678
Item 9	1.175	0.299	3.926	< 0.001	0.498
**Fit indices and standard fit criteria**					
Compliance measures	Good fit	Acceptable fit		Model fit values appropriate to our data structure	
*χ*2		36.857			
Df (p)		26 (*p* = 0.077)			
*χ*2/*d* *f*	0 ≤ *χ*2/df ≤ 2	2 < *χ*2/df ≤ 5		1.418	
TLI	0.95 < TLI ≤ 1.00	0.90 ≤ TLI ≤ 0.95		0.959	
CFI	0.95 < CFI ≤ 1.00	0.90 ≤ CFI ≤ 0.95		0.971	
GFI	0.95 ≤ GFI ≤ 1	0.90 ≤ GFI ≤ 0.95		0.921	
RMSEA	0 ≤ RMSEA ≤ 0.05	0.05 < RMSEA ≤ 0.08		0.063 (0.000–0.107)	

*Note:* Spearman rank correlation coefficient *χ*2: chi‐square.

Abbreviations: CFI, confirmatory fit index; RMSEA, the root mean square error of approximation; RMR, root mean square residuals.

In the subsequent process, model fit was examined through a three‐stage process using goodness‐of‐fit index values. The model‐data fit was checked in three stages using goodness‐of‐fit indices. First, the significance of the chi‐square test statistic (*χ*2) was checked, and based on the obtained value of *χ*2 = 36.857 (*p* = 0.077), it was observed that the model fit the data. In the second stage, the normalized chi‐square (*χ*2/df) value was found to fall within the range of 0 < 1.418 ≤ 2, indicating good fit according to the cutoff points specified in the literature. In the final stage, other goodness‐of‐fit indices were evaluated based on the cutoff points given in the literature. The Tucker–Lewis Index (TLI/RHO) value of 0.959 and the Comparative Fit Index (CFI) value of 0.971 (ranging from 0.95 to 1.00) indicated good fit. The Goodness of Fit Index (GFI) value of 0.921, which falls within the range of 0.90–0.95, indicates a good fit. Additionally, the root mean square error of approximation (RMSEA) value of 0.063 (90% confidence interval: 0.000–0.107), which falls within the acceptable range of 0.05–0.08, also demonstrates acceptable fit [57, 58]. Based on the results obtained in the four stages, the statistical significance of the factor loadings and the good and acceptable fit levels indicated by the goodness‐of‐fit indices support the structural validity of the unidimensional model of the MyFACE scale, as examined through CFA.

### 3.6. Convergent and Divergent Validity

The convergent validity of the MyFACE scale total was assessed by examining the correlation with the NHP, CASP, and PS totals and subdimensions, whereas divergent validity was evaluated by examining the relationship between the PS total and its subdimensions. The results obtained from Table 3 showed that there is convergent validity between the MyFACE scale total and the PS total and subdimensions, as well as between the MyFACE scale total and the CASP total and the neighborhood community participation subdimension. The results showed that there is divergent validity between the MyFACE scale total and the PS total and its subdimensions (Table 3).

### 3.7. Reliability of the Scale

The mean and standard deviation for the scale were found to be (25.05 ± 7.90), and the Cronbach alpha internal consistency coefficient was 0.859. Since Cronbach alpha is not always sufficient or appropriate in all conditions, it is not the only criterion used for reliability estimation [59, 60]. From a factor analysis perspective, McDonald′s (*ω*) alpha coefficient was used, and the McDonald alpha for the unidimensional structure was found to be 0.863. The reliability coefficients obtained were above the 0.70 cutoff point specified by Nunnally and Bernstein (1994), indicating that the measurements obtained from the MyFACE scale are reliable [48].

### 3.8. Test‐Retest Reliability

When examining Table 4, the mean and standard deviation values for the first test and retest for the unidimensional structure were as follows: 25.64 ± 8.06 (min–max: 1–36) and 25.98 ± 7.50 (min–max: 5–36), respectively. The Cronbach alpha values for these tests were 0.894 and 0.887, respectively. When the scale was readministered to 48 participants after a certain period, the ICC was found to be 0.980 (ICC = 0.980, *p* < 0.001). According to Cicchetti′s recommended cutoff, this indicates excellent agreement between the two measurements [52].

**Table 4:** Test‐retest reliability analysis.

Measure	Cronbach′s *α*	*M* *e* *a* *n* ± *S* *D*	ICC (95% CI)	*p*
Test	0.894	25.64 ± 8.06	—	—
Retest	0.887	25.98 ± 7.50	0.980 (0.96–0.99)	< 0.001

Abbreviation: ICC, intraclass correlation coefficient.

The structural validity of the unidimensional model of the MyFACE scale has been confirmed through MRFA and DFA. The reliability of the scale was evaluated using Cronbach alpha (*α*) and McDonald′s alpha (*ω*) coefficients, both of which were above 0.70. Additionally, the test‐retest method showed excellent agreement between the scale′s measurements at two different times. Based on these results, it can be concluded that the MyFACE scale, which consists of a single dimension, is both valid and reliable.

### 3.9. Correlation Between the Total Score of the MyFACE Scale and the Total and Subscale Scores of the NHP, CASP, and PSs: Convergent and Divergent Validity

In Table 5, the convergent validity of the MyFACE total score was evaluated by examining its correlations with the total and subscale scores of the NHP, CASP, and PSs, whereas the divergent validity was assessed by examining the relationships between the total and subscale scores of the PS. The convergent validity between the MyFACE total score and the total and subscale scores of the NHP and CASP scales was examined using the Spearman rank correlation coefficient.

**Table 5:** Correlations between MyFACE total and other measures.

Measure	MyFACE (rho) *ρ*	MyFACE *p*
NHP emotional	0.237	0.015
NHP social	0.351	< 0.001
NHP Part 1 total	0.219	0.025
Participation Subscale 1	−0.299	0.002
Participation Subscale 2	−0.480	< 0.001
Participation total	−0.490	< 0.001
Neighborhood/community participation (CASP)	0.262	0.007
CASP total	0.202	0.039

*Note: ρ* = Spearman′s rank correlation coefficient.

A statistically significant weak positive correlation was found between the MyFACE total score and the NHP Part 1 total score, as well as its emotional and social subdimensions (respectively; rho = 0.219, rho = 0.237, and rho = 0.351) (*p* = 0.025, *p* = 0.015, and *p* < 0.001, respectively). A statistically significant weak positive correlation was found between the MyFACE total score and both the CASP total score and the neighborhood/community participation subdimension (rho = 0.202 and rho = 0.262, respectively) (*p* = 0.039 and *p* = 0.007). The results indicated that there was convergent validity between the MyFACE total score and the total and subscale scores of the PS, as well as between the MyFACE total score and the CASP total score and the neighborhood/community participation subdimension. The divergent validity between the MyFACE total score and the total and subscale scores of the PS was examined using the Spearman rank correlation coefficient. A statistically significant weak negative correlation was found between the MyFACE total score and Participation Subdimension 1 (rho = −0.299, *p* = 0.002). A statistically significant moderate negative correlation was found between the MyFACE total score and both Participation Subdimension 2 and the total score (rho = −0.480 and rho = −0.490, respectively; *p* < 0.001). The results indicated that there was divergent validity between the MyFACE total score and the total and subscale scores of the PS (Table 5).

## 4. Discussion

This study was designed to demonstrate that the MyFACE Scale is a valid and reliable measurement tool for community access and participation. It adds to the body of information from the viewpoint of mothers, bolstering the use of MyFACE as a valid and reliable indicator of community participation and accessibility for families with a disabled child. The scale has demonstrated excellent internal consistency reliability as a whole.

Since 2022, studies on the psychometric properties of the MyFACE assessment have been limited. Bourke‐Taylor et al. used item analysis to explore whether the scale items were discriminative and their consistency with one another while developing the MyFACE scale [3]. In our study, we applied item analysis during the Turkish adaptation of the scale. The item analysis results for the Turkish version showed that all item‐total correlations (ranging from 0.467 to 0.708) were above 0.30, indicating that all items were discriminative. Based on these values, we can conclude that the Turkish version demonstrates that the scale items are integral parts of the whole. No changes were made to the scale based on the item analysis, and the dimensional structure of the 9‐item scale was confirmed. The dimensional structure of the MyFACE scale in the Turkish population has been evaluated using the MRFA method [49]. MRFA is a factor extraction method recommended by Fabrigar et al. and Nunnally and Bernstein [48] (1994) that does not require multivariate normal distribution [61]. During the analysis, polytoric correlation coefficients, which are the most accurate measure for relationships in ordinal variables and do not require multivariate normality, were used for the correlation matrix [62, 63]. The suitability of the dataset for factor analysis was checked using the KMO and Bartlett′s Test of Sphericity. The KMO value was found to be 0.813, indicating that the sample size was adequate. According to the results of Bartlett′s Test of Sphericity (*χ*2 = 567.10, *d* *f* = 36, *p* < 0.001), the correlation matrix of the dataset was significantly different from the identity matrix. Based on these results, it was determined that the dataset is suitable for factor analysis [53].

In the analysis conducted using the FACTOR program, the direct oblimin rotation method [50] was used to obtain a comprehensible and easily interpretable factor structure. The gamma or delta parameter was set to 0 [46]. In the MRFA results, the most commonly used methods in the literature for determining the number of factors are the MAP test, PA, and Kaiser (K1) criterion [51]. Studies related to Kaiser′s (K1) criterion [64], which selects factors with eigenvalues greater than 1, suggest that this method tends to provide more biased estimates and recommends a larger number of factors compared with other methods [65, 66]. For this reason, it is not recommended to use the K1 criterion alone for determining the number of factors [67]. Preacher et al. stated that the concept of the optimal number of factors is a more accurate concept than the true factor number [68]. PA has been shown to be a recommended and frequently used method for deciding the number of factors [69, 70]. Kılıç [70], along with Garrido et al. [71], indicated that MAP analysis provided results closer to the true factor number. Zwick and Velicer [66] reported in their study that MAP and PA methods were the most suitable for determining the number of factors. In light of the information above, when the K1 criterion was used in the FACTOR program, a 2‐factor structure was found [66]. However, when MAP, classical PA [71], and optimal PA [72] methods were applied, all three methods led to the single‐factor structure recommended by Bourke‐Taylor et al. [2].

The statistical significance of the factor loadings and the good fit level of the goodness‐of‐fit indexes supported the construct validity of the one‐dimensional model of the MyFACE scale. A second published study examining the structural validity of MyFACE confirmed its unidimensionality and highlighted the significance of the relationship between healthy maternal behaviors and MyFACE scores [2]. In our study, after confirming the unidimensional structure of the MyFACE scale, we examined its concurrent validity using Spearman correlation analysis. The PedsQL scale was used to examine concurrent validity by Bourke‐Taylor et al. [2]. Their investigation found a favorable and moderate link between the PedsQL Psychosocial Health Score and MyFACE, but not the Physical Health Score. Higher psychosocial problem moms scored lower on MyFACE, which may indicate reduced family engagement. As predicted, moms with children with higher care requirements had more trouble entering the community. In MyFACE, 74% of mothers felt their children′s conduct hindered community involvement, indicating that children′s behavior affects family community participation [2]. Bourke‐Taylor et al. observed a moderate association between mothers′ health and DASS stress, depression, and anxiety [2]. Mothers who did more health‐promoting activities had lower depression and higher MyFACE ratings. MyFACE correlated with maternal health and well‐being characteristics, suggesting that women who feel empowered and have fewer mental health difficulties are more likely to be community‐engaged and regard their families as more active.

In our study, we used the NHP (NSP), PS, and CAPS. A positive and significant correlation was found between MyFACE and NSP. MyFACE also showed a significant positive correlation with CAPS, a measure of the degree of help a child needs to participate in play at home and in the community, as well as with the caregiver′s PS score. These results are consistent with previous studies supporting MyFACE as a tool to assess community accessibility and participation for families of children with disabilities [2, 3]. These findings support family‐centered strategies by acknowledging that family culture affects everyone [73, 74]. In qualitative research, moms of disabled children described how they boosted their leisure time and healthy habits and how their families increased leisure and community involvement [75, 76]. Clinically, these findings suggest that professionals working with children with disabilities and their families will be well‐informed to assess and intervene in order to identify and manage behaviors that affect family participation in the community.

In our study, the reliability of the scale was evaluated using Cronbach′s alpha for internal consistency and the ICC for test‐retest reliability [47, 57, 58]. In the study by Bourke‐Taylor et al., internal consistency reliability was chosen as the method [3]. In our study, data from 105 participants were analyzed, and the total Cronbach′s alpha coefficient of the scale was found to be 0.859. The obtained reliability coefficients were higher than the 0.70 threshold suggested by Nunnally and Bernstein (1994) [48], indicating that the measurements obtained from the “My Family′s Accessibility and Community Engagement (MyFACE)” scale are reliable. In the study by Bourke‐Taylor et al. [3], this value was reported as 0.85. This result shows that the Turkish version exhibits similar and excellent internal consistency to the original version [3].

The test‐retest reliability of the scale was examined to determine its stability over time. The obtained ICC value was 0.980 (*I* *C* *C* = 0.980, *p* < 0.001), indicating excellent agreement between the two measurements, according to the cutoff point recommended by Cicchetti [52]. Clinically, these results imply that practitioners who work with families and children with disabilities will be knowledgeable enough to identify and control behaviors that impact family involvement in the community through assessment and intervention. Identifying the barriers to participation is crucial, particularly in evaluating factors that influence the inclusion of individuals with disabilities and their families [76].

The importance of a child′s capacity to participate in daily activities has been widely accepted and has become a focal point for occupational therapy practitioners [75–78]. In order to increase the child′s involvement in routines and activities at home and in the community, parent–child interventions have been gaining traction recently [79]. For occupational therapists, the common goal is to successfully enhance participation by considering all these factors [80]. In order to increase children′s participation in activities, occupational therapists should conduct detailed assessments of the child, environment, and activity to identify the factors influencing community participation. Based on these assessments, a collaborative approach should be planned to facilitate the child′s and family′s participation in the activities they have identified [67]. When planning intervention programs and re‐evaluating families after implementation, it is especially important to consider how children and families rate their own lives, functions, and well‐being. Barriers like parental health and exhaustion, perceived stigma, physical inaccessibility, lack of skill assistance, transportation problems, and time constraints are easily identified by MyFACE. Bourke‐Taylor et al. highlight that MyFACE is a significant family‐centered, brief self‐report tool for uncovering families′ perceptions of all barriers to community accessibility and participation [2, 3, 81]. This tool plays a crucial role in service delivery for families raising children with disabilities, offering insights into the challenges they face in accessing and participating in the community.

### 4.1. Implications for Practice and Training

The results of this study in terms of occupational therapy applications are as follows:

• By providing occupational therapists with a validated tool to assess the community accessibility and involvement of families raising children with impairments, the Turkish version of the MyFACE Tool facilitates data‐driven therapies.
• Therapists can encourage everyone to take part by making community‐based interventions that are more culturally appropriate. To do this, they need to be aware of the barriers and facilitators in the Turkish setting.
• The tool enables clinicians to comprehend parental perceptions of accessibility more effectively, enabling them to advocate for services and policies that improve families′ well‐being and resilience.
• The validated tool contributes to occupational therapy research by providing standardized data that can inform policy decisions and improve accessibility initiatives for individuals with disabilities and their families.

### 4.2. Limitations and Strengths

The fact that the study was limited to a single center and no comparisons were made with other institutions constitutes some of the limitations of the research. Although the sample size was sufficient for statistical analysis, it may restrict the generalizability of the study′s findings.

The Turkish validity and reliability study of MyFACE significantly advances the existing knowledge on the tool by demonstrating its applicability across different cultural and linguistic contexts. The validation of the scale in Turkish not only confirms the robustness of the MyFACE structure but also supports its suitability for evaluating the community participation of children with disabilities and their families, thereby expanding its use in international research. Furthermore, the study provides insights into the linguistic and conceptual differences that may arise when adapting participation measures for non‐English‐speaking communities, highlighting the importance of cultural sensitivity in psychometric evaluations. This research not only strengthens the reliability of MyFACE within the Turkish context but also serves as a model for further cultural adaptations necessary for comprehensive international studies.

The Turkish validity and reliability study of MyFACE also offers several practical recommendations for its use in family participation contexts. First, the Turkish MyFACE scale provides families with a valid and effective tool for assessing and exploring the relationship between local community accessibility and the participation of children with disabilities. In addition, the Turkish version of MyFACE can help families identify areas of participation that need improvement and evaluate the outcomes of interventions aimed at enhancing participation.

The study also emphasizes the importance of involving families in the adaptation process and suggests that institutions should consider ongoing feedback from families. Finally, the Turkish version of MyFACE can be used in comparative studies with other cultural adaptations of the scale, providing valuable insights for communities seeking to understand family participation trends across different regions. This approach not only supports broader intervention strategies but also contributes to community participation—an essential aspect of everyday life. In particular, during the preschool period, when family participation is crucial, valuable information can be obtained to help teachers, school administrators, and families provide the necessary support for children. Moreover, conducting validity and reliability studies of the scale on various samples is of great importance. Further studies using MyFACE will enhance its measurement power and establish it as a valuable assessment tool for advancing research on family participation. The limitations of this research include the insufficient sample size for examining other types of validity, such as structural validity, and the constraints in investigating criterion validity due to the lack of comparable instruments to MyFACE. Overall, a larger sample size would allow for a more in‐depth evaluation of psychometric properties such as structural validity, criterion validity, reliability, and measurement error.

MyFACE is an important family‐centered, brief self‐report tool that helps families identify all barriers they encounter in community accessibility and participation. This tool plays a critical role in service delivery for families raising children with disabilities by providing valuable insights into the challenges they face in accessing and participating in community life.

## 5. Conclusion

This study established the validity and reliability of the Turkish version of the MyFACE tool, which provides a standardized tool to assess community participation and accessibility for families raising children with disabilities. The Turkish version of MyFACE is a brief and psychometrically reliable tool for allied health professionals who aim to increase community participation of children with disabilities and their families. The availability of a culturally adapted assessment tool will support occupational therapists, researchers, and policymakers in developing targeted interventions to promote inclusive participation. Further studies should investigate the applicability of the tool in different settings and its effectiveness in guiding intervention strategies.

## Author Contributions

**Ayşe Göktaş**: writing – review & editing, writing – original draft, validation, methodology, investigation, data curation, conceptualization. **Esma Özkan:** writing – review & editing, validation, methodology. **Tuana Harman:** investigation, data curation, methodology.

## Funding

No funding was received for this manuscript.

## Ethics Statement

The study group was obtained from Health Sciences University, non‐Interventional Clinical Research Ethics Committee (ethical date 16.05. 2023, Decided Number 2023‐184).

## Consent

Written informed consent was obtained from each participant prior to their participation in the interview.

## Conflicts of Interest

The authors declare no conflicts of interest.

## Data Availability

The datasets utilized and/or analyzed during the present study are available from the corresponding author upon reasonable request.
